# End group functionalization of poly(ethylene glycol) with phenolphthalein: towards star-shaped polymers based on supramolecular interactions

**DOI:** 10.3762/bjoc.10.235

**Published:** 2014-09-25

**Authors:** Carolin Fleischmann, Hendrik Wöhlk, Helmut Ritter

**Affiliations:** 1 Institute of Organic Chemistry and Macromolecular Chemistry, Heinrich-Heine-University Düsseldorf, Universitätsstraße 1, D-40225 Düsseldorf, Germany

**Keywords:** cyclodextrin, phenolphthalein, poly(ethylene glycol), supramolecular assembly, UV–vis spectroscopy

## Abstract

The synthesis of a new phenolphthalein azide derivative, which can be easily utilized in polymer analogous reactions, is presented. The subsequent cycloaddition reaction with propargyl-functionalized methoxypoly(ethylene glycol) yielded polymers bearing phenolphthalein as the covalently attached end group. In presence of per-β-cyclodextrin-dipentaerythritol, the formation of stable inclusion complexes was observed, representing an interesting approach towards the formation of star shaped polymers. The decolorization of a basic polymer solution caused by the complexation was of great advantage since this behavior enabled following the complex formation by UV–vis spectroscopy and even the naked eye.

## Introduction

Over the past decades, polymers with well-defined and complex architectures gained increasing attention due to a broad variety of applications. Accordingly, the development of new synthetic routes to these polymeric structures is still of great interest [1–5]. A very convenient approach in the synthesis of such systems is the attachment of compounds indicating the successful formation of the desired architecture, e.g., by a color change [6–7].

Compared to analogous linear systems, dendrimeric and hyperbranched polymers offer different and virtually unusual properties such as lower viscosities [8], a higher solubility due to numerous end groups [9], and a rather globular shape instead of entangled polymer chains [10–11]. Applications deriving therefrom include the use as nanocarriers [12–13], electro-optical materials [14], coating additives [15] and rheology modifiers [16].

The link between hyperbranched polymeric materials and linear polymers is represented by star-shaped polymers. This interesting class of polymer architectures can be subdivided into regular star polymers and miktoarm star polymers. In analogy to hyperbranched polymers, star shaped polymers have lower viscosities than analogous linear materials of the same molecular weight because the viscosity is rather determined by the mass of one arm than the mass of the whole molecule [17].

Miktoarm star polymers additionally offer complementary properties as they are built of different polymeric arms [18–24].

The most common techniques applied in the preparation of these compounds are controlled/living polymerizations. While several decades ago, anionic polymerization was the method of choice [25–28], in recent years, controlled radical polymerizations (CRP) gained increasing importance and nowadays offer a variety of synthetic routes to well-defined polymeric structures [29–33]. A very interesting alternative is the combination of CRP with supramolecular complex formation, e.g., by the use of cyclodextrins (CDs) [6–7,34].

The most important representatives of these cyclic oligosaccharides with respect to industrial applications consist of six (α-CD), seven (β-CD) and eight (γ-CD) glucopyranose units and have a cone-like structure which is hydrophilic on the outside and rather hydrophobic on the inside [35]. The ether linkages of the glucose units are located on the inside of the cavity which induces a high electron density and enables the complexation of suitable hydrophobic guest molecules and thus, offers a broad variety of applications [36–39]. An adequate guest molecule for β-CD is the well-known indicator dye phenolphthalein [40]. In addition to its relatively high affinity to β-CD, this molecule undergoes a decolorization in basic solution as the complexation induces a re-lactonization of the molecule without protonation of the phenolic hydroxy groups [41]. Therefore, phenolphthalein can be utilized as an indicator for the formation of supramolecular complexes with β-CD [42–44]. Recently, we transferred this principle to phenolphthalein that was covalently attached to several polymers [7,45–46].

In this study, we present a new approach towards the preparation of star-shaped polymers that are formed through supramolecular interactions of β-CD and phenolphthalein. The great advantage of this system would be the possibility to follow the star polymer formation with naked eyes.

## Results and Discussion

We herein describe the synthesis of the host and guest compound, which were further utilized for the preparation of star shaped polymers based on supramolecular interactions.

The preparation of both, the host and the guest compound, occurred via 1,3-dipolar cycloaddition, in which the cyclodextrin (CD) and phenolphthalein (PP) moieties were introduced as the complex-forming groups. Regarding the formation of inclusion complexes, one of the major driving forces is the displacement of enthalpy-rich water molecules from the cyclodextrin cavity, which requires working in aqueous media, ideally in pure water. With the intention to enhance the guest molecule’s hydrophilicity in order to transfer it into the aqueous phase, poly(ethylene glycol) was chosen as the PP-bearing molecule. On the other hand, it was also of great importance to maintain the rather hydrophobic nature of the PP moiety, since this nature strongly supports the inclusion complex formation. With respect to both requirements, methoxypoly(ethylene glycol) with an average molecular weight of 350 g/mol was chosen, which should generally allow working in aqueous media while preserving the hydrophobic nature of the PP moiety. The resulting molecules, namely the phenolphthalein end group-modified methoxypoly(ethylene glycol) (**mPEG-PP**) and the dipentaerythritol derivative per-β-cyclodextrin-dipentaerythritol (**DPE-CD**), decorated with six cyclodextrin moieties, were investigated with respect to their complexation behavior.

### Synthesis of host and guest compounds

The preparation of methoxypoly(ethylene glycol) (mPEG)-bearing phenolphthalein as a functional end group proceeded through a 1,3-diploar cycloaddition reaction of alkyne functionalized mPEG (**mPEG-prop**) and an azide-functionalized phenolphthalein (**PP-N_3_**) derivative. The synthesis of the **PP-N_3_** intermediate was of special interest, since up to now this molecule has not been described in literature and can be employed in various reactions aiming for the (end) group functionalization of polymers.

**Scheme 1:**
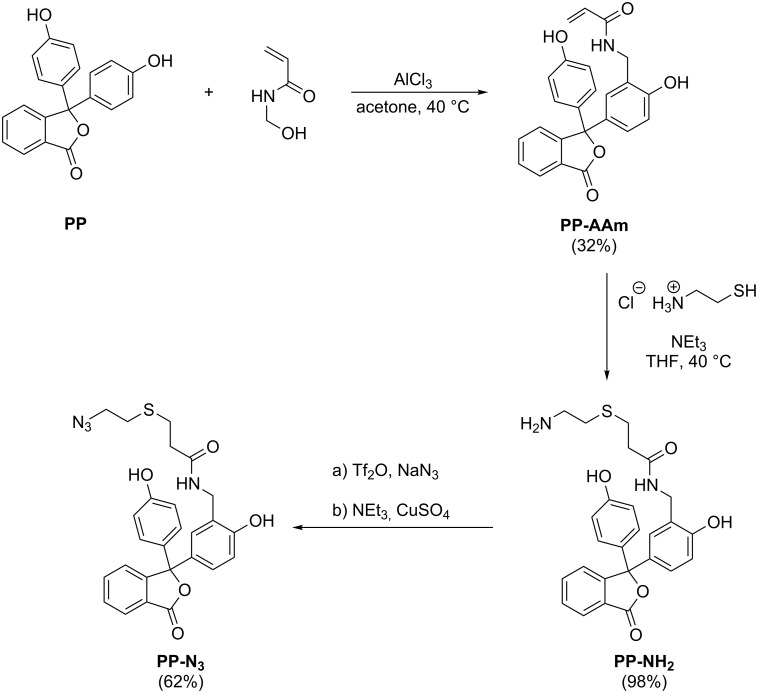
Three-step synthesis of azide-functionalized phenolphthalein derivative **PP-N_3_**. a) H_2_O, CH_2_Cl_2_, 0 °C. b) H_2_O, MeOH, CH_2_Cl_2_, room temperature.

The molecule was prepared in a three-step reaction starting from native phenolphthalein (see Scheme 1).

In a first step, the monofunctional phenolphthalein-monomer *N*-(2-hydroxy-5-(1-(4-hydroxyphenyl)-3-oxo-1,3-dihydroisobenzofuran-1-yl)benzyl)acrylamide (**PP-AAm**) was synthesized following a protocol we developed in a previous study [46]. Afterwards, the corresponding amine derivative (**PP-NH_2_**) was obtained through a Michael addition reaction of **PP-AAm** and cysteamine hydrochloride. The hydrochloride was chosen in order to disable the competing nucleophilic addition of the amine and therefore, to prevent the formation of side products. The purified **PP-NH_2_** was then converted into the corresponding azide derivative (**PP-N_3_**) by use of trifluoromethanesulfonyl azide, which was generated in situ from sodium azide and trifluoromethanesulfonic anhydride as described in literature [47]. Subsequent treatment with **PP-NH_2_** yielded the desired product **PP-N_3_** in an overall yield of 19%.

**Scheme 2:**
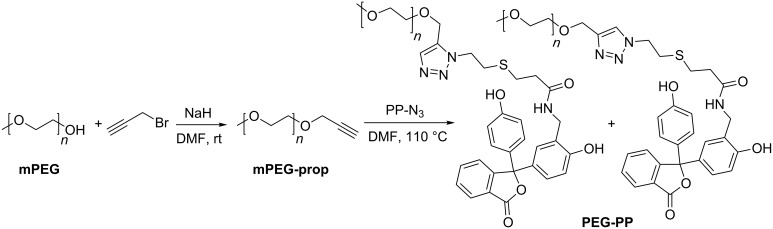
Synthesis of the dipolarophil **mPEG-prop** and subsequent coupling with **PP-N_3_**.

The dipolarophil was prepared starting from methoxypoly(ethylene glycol) (**mPEG**) and propargyl bromide, whilst the latter one was utilized in a threefold excess to achieve maximum conversion of the starting material. The resulting alkyne functionalized mPEG α-methoxyethyl-ω-propargyloxypoly(ethylene glycol) (**mPEG-prop**) was then reacted with **PP-N_3_** in a 1,3-diploar cycloaddition, which gave the desired phenolphthalein-functionalized **mPEG-PP** (see Scheme 2). Since the reaction was carried out in absence of copper(I) salts, a mixture of the 1,4- and 1,5-substituted triazole regioisomers was obtained.

The host component based on dipentaerythritol, which was converted into a multifunctional initiator for core first star polymer preparation approaches in previous studies [48–49], was synthesized according to a protocol previously developed in our group [50]. Dipentaerythritol was functionalized with six propargyl moieties serving as the dipolarophil in a subsequent treatment with β-cyclodextrin azide. Thereby, a dipentaerythritol derivative carrying six cyclodextrins (**DPE-CD**) that are covalently attached through triazole rings was obtained.

**Figure 1:**
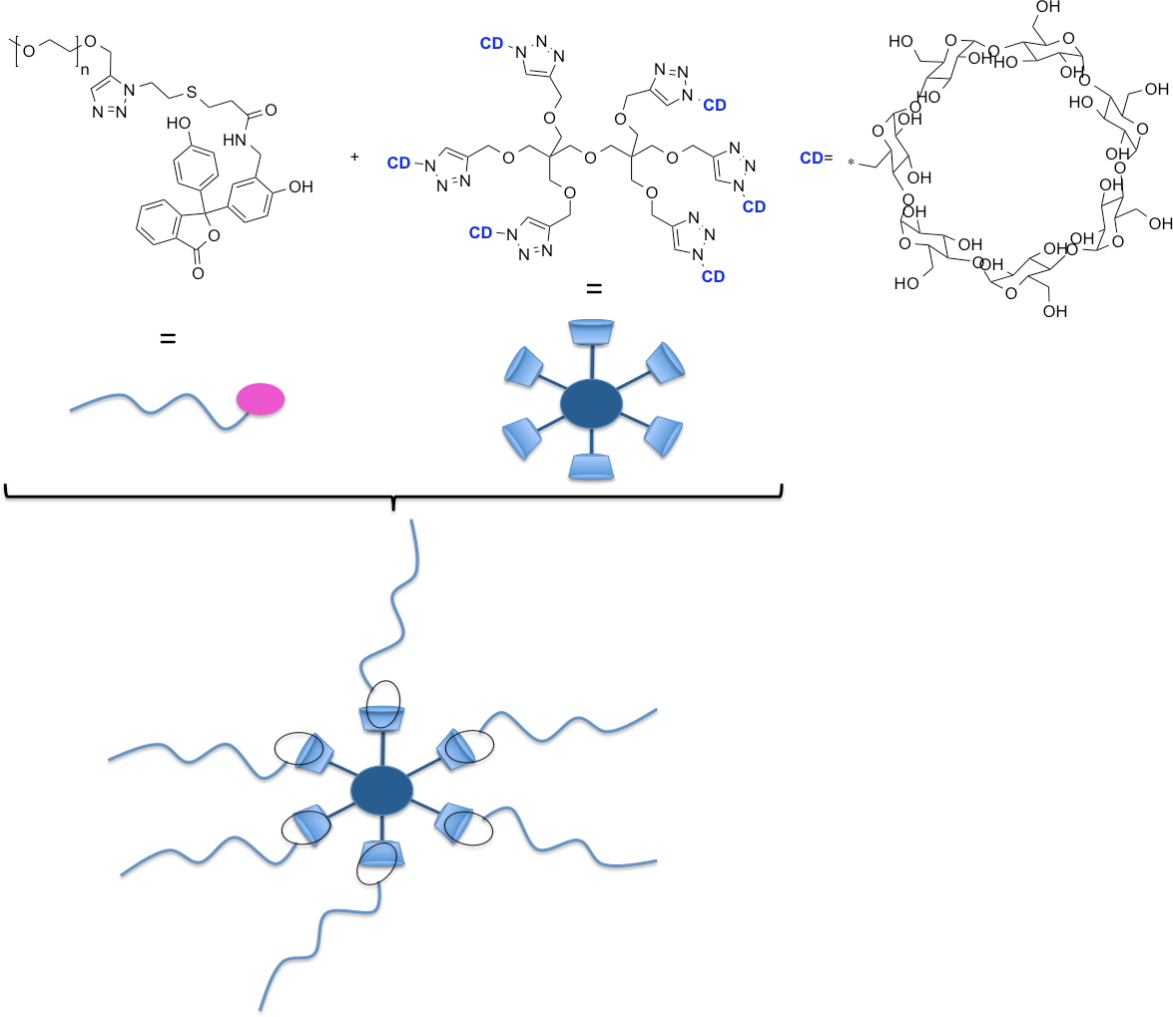
Schematic illustration of the complex formation of **PEG-PP** and **DPE-CD**.

Mixing of **mPEG-PP** and **DPE-CD** resulted in the formation of stable complexes (see Figure 1), which were subjected to further investigations.

### Investigation of the complex formation behavior

Due to the fact that in basic solution the complexation of PP by β-CD is accompanied with a decolorization due to the re-lactonization of the molecule, a first evaluation of the complex formation occurred with bare eyes.

**Figure 2:**
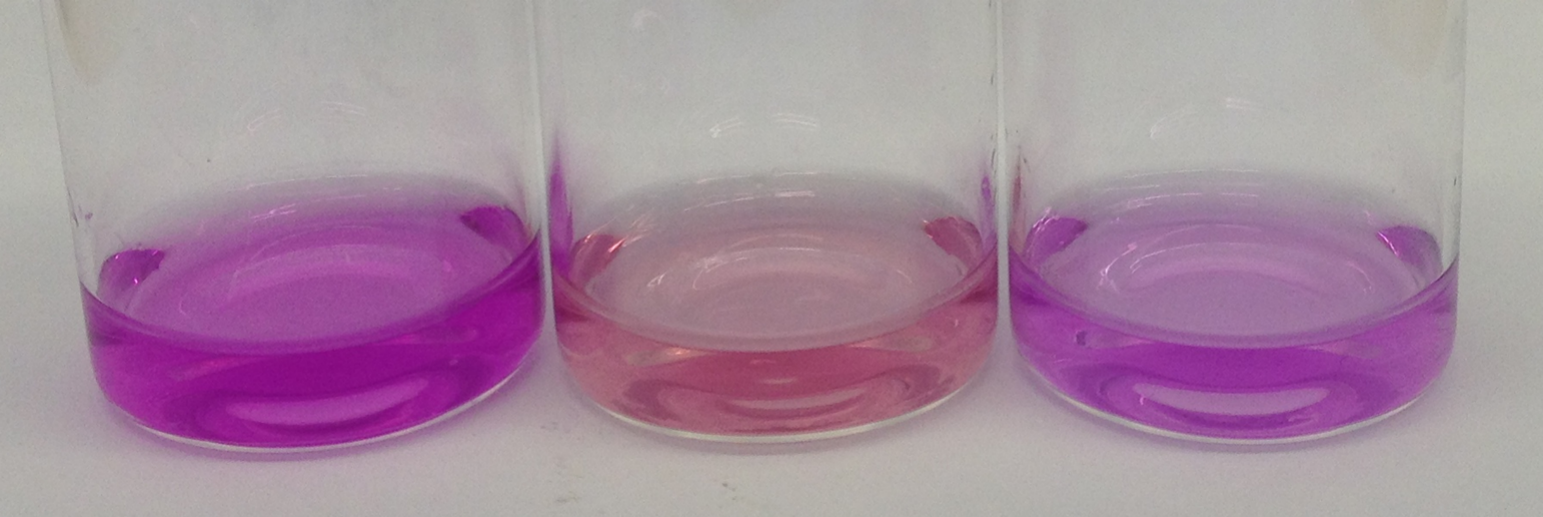
Solution of **PEG-PP** (0.05 mg/mL) a) at pH 10, b) in presence of 16.7 equiv **DPE-CD** at pH 12, c) in presence of 100 equiv RAMEB-CD at pH 12 (from left to right).

For this, a molar excess of 16.7 **DPE-CD** equivalents, which equals 100 CD units per PP moiety, was added to a solution of 0.05 mg/mL PEG-PP at pH 12. In order to compare the complexation ability of **DPE-CD** to free β-CD, a sample containing the same number of equivalents of monomeric, randomly methylated β-cyclodextrin (RAMEB-CD) was prepared. For both samples, a distinct decolorization was observed, although a complete decolorization did not occur (see Figure 2).

As an explanation of this behavior we assumed that over time, an equilibrium of complexed and decomplexed molecules is reached in which the high extinction coefficient of the non-lactonic, decomplexed PP molecules causes the residual colorization. This assumption was supported by the observation that the addition of higher amounts of cyclodextrin equivalents, which should shift the equilibrium to the complexed form, indeed resulted in a stronger decrease of the colorization.

**Figure 3:**
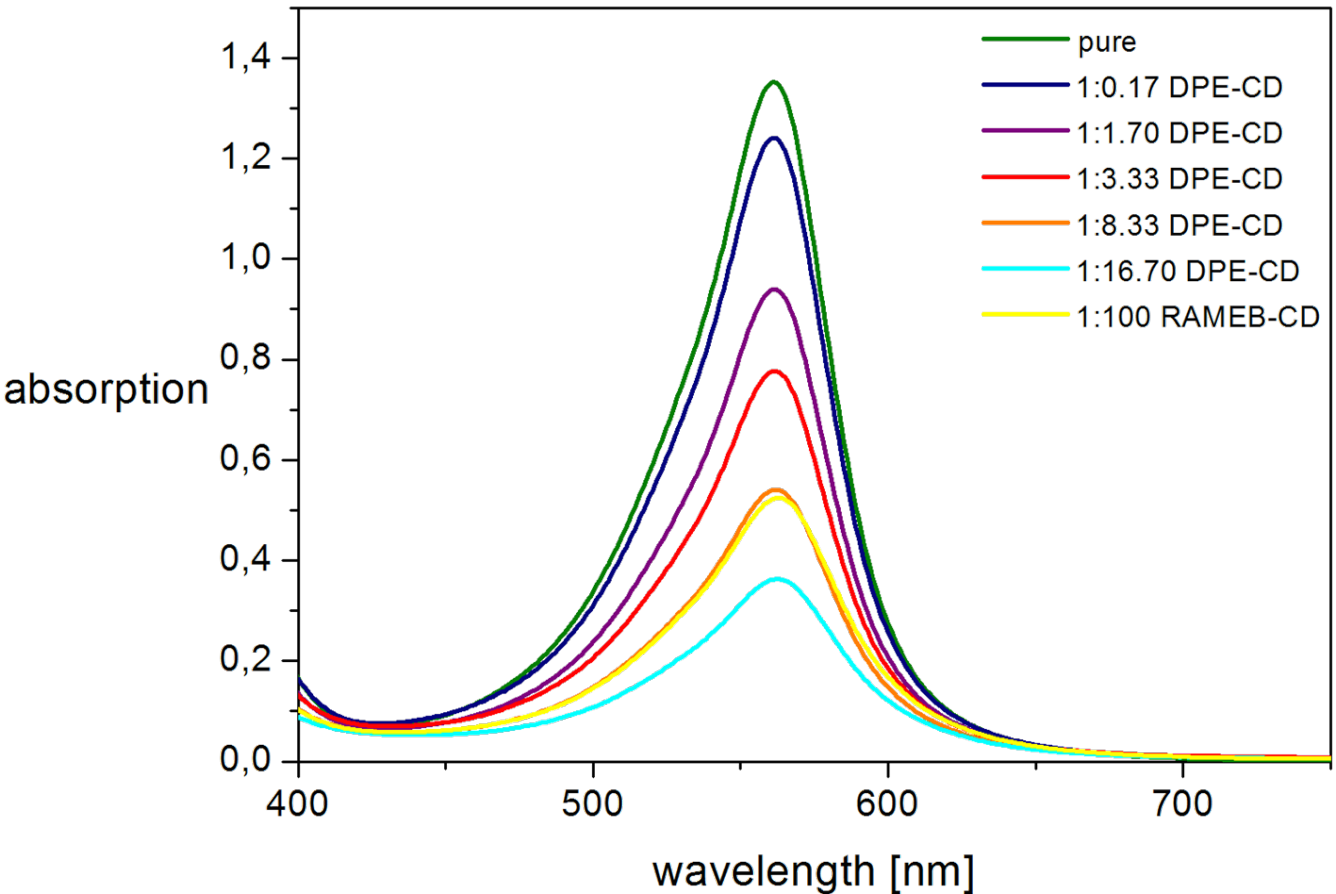
UV–vis spectra of **PEG-PP** solutions containing different amounts of **DPE-CD** and **RAMEB-CD**.

In addition to the qualitative evaluation of the complexation behavior of **PEG-PP**, UV–vis measurements were performed in order to get an insight into the quantitative complex analysis. For native phenolphthalein, the characteristic absorption maximum that refers to the pink color in basic solution can be found at 554 nm in corresponding UV–vis spectra. For the phenolphthalein-containing polymer **PEG-PP**, a slight bathochromic effect is observed, which shifts the maximum to 561 nm. Accordingly, the decrease of the absorption at 561 nm was examined in order to evaluate the complexation. For this, solutions containing a **PEG-PP**:**DPE-CD** ratio between 1:1 and 1:16.7, and a **PEG-PP**:**RAMEB-CD** ratio of 1:100, respectively, were measured at pH 12. From the corresponding spectra in Figure 3, it can be seen that the absorption at 561 nm clearly decreases with increasing amount of **DPE-CD**. Interestingly, the complexation with **DPE-CD** seems to be more efficient than the complexation by free RAMEB-CD, since the presence of 100 equivalents β-CD attached to DPE induces a stronger decrease in the absorption than the same amount of free β-CDs. A similar effect was observed in a previous study in which the complexation of polymer-bound phenolphthalein by a β-CD-terminated polymer was investigated [7]. We assume that this behavior can be attributed to entropic effects.

In addition to the UV–vis measurements, which already verified the formation of supramolecular complexes, rotating-frame nuclear Overhauser effect correlation spectroscopy (ROESY) was performed. Thereby, resonances of spatially close nuclei are connected though cross peaks which can be found in ROESY spectra besides the diagonal peaks. Since the complex formation of **DPE-CD** and **PEG-PP** induces a spatial convergence of the PP and β-CD moieties, we expected the detection of cross peaks between the CD protons and the aromatic PP protons. Due to the limited solubility of **PEG-PP** in aqueous solution, a sample of low polymer concentration was utilized to record the ROESY spectrum.

In the corresponding spectrum, cross peaks of weak intensity were found (see Figure S1, Supporting Information File 1). Although the low intensity can most likely be referred to the low polymer concentration, the significance of this experiment needs to be critically evaluated.

The formation of the desired star-shaped polymers requires the complexation of **PEG-PP** molecules by at least three β-CD moieties attached to the same **DPE-CD** molecule. We assumed that the complexation efficiency of **DPE-CD** can be evaluated by comparison of the hydrodynamic diameters of free **DPE-CD** and the diameter of the complex formed by both polymers. Ideally, the hydrodynamic diameter of the complex could be calculated by the following equation:

[1]
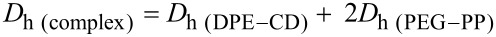


Unfortunately, neither the hydrodynamic diameters of the single polymers nor the diameter of the complex could be determined in DLS measurements. Instead, a strong aggregation behavior was observed and dilution or filtration resulted in a dramatic decrease of the measurement quality.

## Conclusion

A successful synthetic strategy for the preparation of new compounds bearing phenolphthalein and cyclodextrin moieties as the complex forming groups could be introduced. Furthermore, the formation of stable complexes based on supramolecular interactions between both compounds was proved by UV–vis measurements. Although DLS measurements did not succeed to explicitly validate the formation of the desired star shaped polymers due to the formation of strongly aggregated particles, we believe that this approach is an interesting addition to other techniques aiming for the preparation of star shaped polymers based on supramolecular interactions.

Additionally, with respect to the importance of phenolphthalein in analytical procedures, the synthetic route developed for the preparation of phenolphthalein terminated poly(ethylene glycol) should be of great interest for future investigations and applications, since this synthetic route enables the efficient end group modification of polymers with a phenolphthalein moiety.

## Supporting Information

File 1 Detailed experimental procedures and spectroscopic data of the reaction products.
