# Supplementary material for: End group functionalization of poly(ethylene glycol) with phenolphthalein: towards star-shaped polymers based on supramolecular interactions
Source: Beilstein J Org Chem. 2014 Sep 25;10:2263–9. doi: 10.3762/bjoc.10.235 (PMC4187025; doi:10.3762/bjoc.10.235)
Supplement: File 1 — Detailed experimental procedures and spectroscopic data of the reaction products. [file Beilstein_J_Org_Chem-10-2263-s001.pdf]

Supporting Information

for

End group functionalization of poly(ethylene glycol) with phenolphthalein: towards star-shaped polymers based on supramolecular interactions

Carolin Fleischmann, Hendrik Wöhlk, Helmut Ritter*

Address: Institute of Organic Chemistry and Macromolecular Chemistry, Heinrich-Heine-University Düsseldorf, Universitätsstraße 1, D-40225 Düsseldorf, Germany

Email: Helmut Ritter - h.ritter@hhu.de
* Corresponding author

Detailed experimental procedures and spectroscopic data of the reaction products

Table of contents

1 Materials 2
2 Measurements 2
3 Syntheses 3
3.1 Synthesis of N-(2-hydroxy-5-(1-(4-hydroxyphenyl)-3-oxo-1,3-dihydroisobenzofuran-1-yl)benzyl)acrylamide (PP-AAm) 3
3.2 Synthesis of 3-((2-aminoethyl)thio)-N-(2-hydroxy-5-(1-(4-hydroxyphenyl)-3-oxo-1,3-dihydroisobenzofuran-1-yl)benzyl)propanamide (PP-NH_2) 4
3.3 Synthesis of 3-((2-azidoethyl)thio)-N-(2-hydroxy-5-(1-(4-hydroxyphenyl)-3-oxo-1,3-dihydroisobenzofuran-1-yl)benzyl)propanamide (PP-N_3) 4
3.4 Synthesis of α-methoxyethyl-ω-propargyloxypoly(ethylene glycol) (mPEG-prop) 5
3.5 Synthesis of PEG-PP 5
3.6 Synthesis of 3-(3-(3-(prop-2-inyloxy)-2,2-bis((prop-2-inyloxy)methyl)propoxy)- 2,2-bis((prop-2-ynyloxy)methyl)propoxy)prop-1-yne [2] 6
3.7 Synthesis of mono-6^I-tosyl-6^I-desoxy-β-cyclodextrin [2] 6
3.8 Synthesis of mono-6^I-azido-6^I-desoxy-β-cyclodextrin [2] 7
3.9 Synthesis of per-β-CD-dipentaerythritol (DPE-CD) [2] 7
4 ROESY spectrum of the PEG-PP–DPE-CD complex 8
5 References 8

1 Materials

N-(hydroxymethyl acrylamide) (TCI Europe, ≥98%), aluminum chloride (Riedel-de-Haën, ≥98%), phenothiazine (Sigma Aldrich, 98%), phenolphthalein (Carl Roth GmbH, >99%), magnesium sulfate (Acros Organics, 99%, extra pure), cysteamine hydrochloride (Appli Chem, techn.), triethylamine (Appli Chem, techn.), sodium azide (Acros Organics, 99%), trifluoromethanesulfonic anhydride (Sigma Aldrich, ≥ 99%), sodium hydrogen carbonate (Grüssing, 99%), copper(II) sulfate pentahydrate (Fluka, p.a.), sodium chloride (Fisher Chemical, techn.) , methoxypoly(ethylene glycol) (Sigma Aldrich, M_n = 350 g/mol, %), sodium hydride (Sigma Aldrich, 60 wt % dispersion in petroleum), propargyl bromide (Sigma Aldrich, 80 wt% solution in toluene), sodium hydroxide (Appli Chem , p.a.) were used without further purification.

α–, β– und γ-cyclodextrins were purchased from Wacker Chemie in pharmaceutical pure grade. The compounds were stored over Sicapent® in a desiccator.

For purification, 5 g of dipentaerithritol (Sigma-Aldrich, techn.) were dissolved in 80 mL hot water and 150 mL methanol and filtered over aluminum oxide (pH7) while still hot. The precipitate formed during cooling down was collected and dried in vacuum. The pure product was then stored over Sicapent® in a desiccator.

All solvents were used in analytically pure grades. *N,N*-Dimethylformamide was purchased in extra dry grade (Acros Organics, 99.8%) and additionally dried over molecular sieve prior to use.

Thin layer chromatography (TLC) was performed on aluminum sheets coated with silica gel 60 F_{254} (Merck). Silica gel (Acros Organics, ultra pure, 40–60 nm, 60A) was employed for column chromatography.

2 Measurements

^{1}H-NMR spectroscopy was conducted on a Bruker Avance III 300 (300 MHz) and a Bruker AC 500 (500 MHz) at room temperature. Dimethyl sulfoxide-d_6 (Deutero GmbH, 99.9%) was used as internal standard. ROESY spectra were recorded on a Bruker Avance III 600 (600 MHz) at room temperature.

MALDI–TOF measurements were performed on a MALDI–TOF Ultraflex I (Bruker Daltonics).

ESI mass spectra were recorded with a mass spectrometer type Ion-Trap-API Finnigan LCQ Deca.

FTIR spectra were recorded on a Nicolet ATR-FTIR spectrometer type FT-IR-6700.

Elementary analyses were performed with a Perkin Elmer Series II Analyser 2400.

UV–vis spectroscopic measurements were performed using a dual-trace spectrometer Specord® 210 Plus from Analytik Jena AG. The solutions were placed in a quartz cuvette (d = 1 cm) and the measurements were performed at room temperature between 400 and 750 nm. All measurements were performed at a final polymer concentration of 0.05 mg/mL, for this, a stock solution containing 0.1 mg/mL PEG-PP was prepared and mixed

in a 1:1 (v/v) ratio with a solution containing the complexing agent. Therefore, the solution of the complexing agent was prepared at a concentration twice as high as the desired final concentration. All solutions were prepared by use of distilled water adjusted to a pH of 12 by use of sodium hydroxide.

Example:
Desired concentration: 0.05 mg/mL PEG-PP + 1 equivalent CD per PEG-PP

- 0.05 mg/mL PEG-PP = $5.15 \cdot 10^{-5}$mmol/mL
- 6 CD units per DPE-CD → $8.59 \cdot 10^{-6}$mmol/mL= 0.06 mg/mL

→ A solution containing 0.1 mg/mL PEG-PP and a solution containing 0.12 mg/mL DPE-CD were prepared and mixed in a 1:1 (v:v) ratio.

DLS data were recorded in backscattering mode on a Malvern Zetasizer Nano ZS ZEN 3600 at a temperature of 25 °C with a laser wavelength of 633 nm and a detection angle of 173°. The solutions were placed in a glass cuvette with a layer thickness of 1 cm. The non-negative-least-squares algorithm in the general-purpose mode was used for interpretation. Each experiment was performed at least 5 times (13–15 runs for each measurement, run duration 10 s) to obtain statistical information and the number-averaged diameters were used for interpretation.

Flash chromatography was performed using a Combi Flash Companion from Teledyne Isco, Inc.

Microwave-assisted syntheses were conducted in a laboratory microwave Discover® 1 by CEM.
The microwave works at a frequency of 2.45 GHz and the maximum power available is 300 W. The experiments were supervised through a coupled temperature and pressure sensor (max. pressure: 20 bar). An IR sensor was employed as the temperature sensor. The reactions were performed in pressure resistant test tubes.

Freeze drying was performed with a construction by Christ, type 1-4 LD plus having a capacity of 4 kg and an ice condenser temperature of −42 °C. The vacuum of 0.1 mbar was achieved by application of a rotary vane pump by Vacuubrand, type RZ6.

3 Syntheses

3.1 Synthesis of *N*-(2-hydroxy-5-(1-(4-hydroxyphenyl)-3-oxo-1,3-dihydroisobenzofuran-1-yl)benzyl)acrylamide (PP-AAm)

The synthesis of PP-AAm was performed following a protocol developed in a previous study [1].

3.2 Synthesis of 3-((2-aminoethyl)thio)-*N*-(2-hydroxy-5-(1-(4-hydroxyphenyl)-3-oxo-1,3-dihydroisobenzofuran-1-yl)benzyl)propanamide (PP-NH_2)

N-(2-hydroxy-5-(1-(4-hydroxyphenyl)-3-oxo-1,3-dihydroisobenzofuran-1-yl)benzyl)acrylamide (**PP-AAm**) (803 mg, 2.00 mmol) and cysteamine hydrochloride (227 mg, 2.00 mmol) were placed in a round bottom flask. Triethylamine (28 µL, 0.20 mmol) and 1.6 mL tetrahydrofuran were added and the resulting solution was stirred at 40 °C overnight. The solvent was removed in vacuum and the crude product was purified by column chromatography (ethyl acetate/dichloromethane 1:1, followed by ethanol/acetone 1:1).

Yield: 937 mg (1.96 mmol, 98%)

^{1}H NMR (DMSO-d_6, 300 MHz): δ [ppm]= 8.41 (t, ^{3}J= 5.8 Hz, 1H), 7.89 (m, 1H), 7.81 (m, 1H), 7.75-7.58 (m, 2H), 7.22-6.93 (m, 4H), 6.92-6.71 (m, 3H), 4.14 (d, ^{3}J= 5.6 Hz, 2H), 2.87 (t, ^{3}J= 7.0 Hz, 2H), 2.77-2.52 (m, 4H), 2.35 (t, ^{3}J= 7.3 Hz, 2H).
FTIR [cm^{-1}]: 1737 (s), 1633 (m), 1610 (m), 1550 (m), 1510 (s), 1253, 1107, 929, 831, 755, 693, 590.
ESI-MS (m/z (%)): 479 $[M + H]^+$ (100).

3.3 Synthesis of 3-((2-azidoethyl)thio)-*N*-(2-hydroxy-5-(1-(4-hydroxyphenyl)-3-oxo-1,3-dihydroisobenzofuran-1-yl)benzyl)propanamide (PP-N_3)

Sodium azide (1.75 g, 26.9 mmol), 4 mL water and 2 mL dichloromethane were placed in a round bottom flask and cooled to 0 °C. Trifluoromethanesulfonic anhydride (0.75 mL, 4.55 mmol) was added drop wise and the resulting mixture was stirred at 0 °C for two hours.
The aqueous phase was then extracted with 3 mL dichloromethane (3 x), and the combined organic layers were washed with saturated sodium hydrogen carbonate solution.

The organic phase was then slowly added to a second flask containing a solution of 3-((2-aminoethyl)thio)-*N*-(2-hydroxy-5-(1-(4-hydroxyphenyl)-3-oxo-1,3-dihydroisobenzofuran-1-yl)benzyl)propanamide (**PP-NH_2**) (773 mg, 1.62 mmol), copper(II) sulfate pentahydrate (19 mg, 0.08 mmol), triethylamine (624 µL, 4.50 mmol) in 1 mL dichloromethane, 0.25 mL water and 1.5 mL methanol. The mixture was stirred at room temperature overnight.
The reaction mixture was then washed with 15 mL saturated sodium hydrogen carbonate solution and extracted with 15 mL dichloromethane (3x). The combined organic layers were then washed with brine, dried with magnesium sulfate and the solvent removed in vacuum. The crude product was purified by column chromatography (acetone/dichloromethane 1:2).

Yield: 10 mg (1.01 mmol, 62%)

^1H NMR (DMSO-d_6, 300 MHz): δ [ppm]= 9.86 (s, 1H), 9.64 (s, 1H), 8.32 (t, ^3J= 5.8 Hz, 1H), 7.89 (m, 1H), 7.81 (m, 1H), 7.76-7.58 (m, 2H), 7.18-6.93 (m, 4H), 6.83-6.69 (m, 3H), 4,15 (d, ^3J= 5.8 Hz, 2H), 3.46 (t, ^3J= 6.8 Hz, 2H), 2.83-2.52 (m, 4H), 2.35 (t, ^3J= 7.3 Hz, 2H).
FTIR [cm^{-1}]: 2102 (m), 1736 (s), 1632(m,), 1610 (m), 1541 (m), 1512 (s), 1252, 1106, 931, 831, 755, 693, 589.
ESI-MS (m/z (%)): 505 $[M + H]^+$ (100).
Elementary analysis: Calculated: 61.89% C; 4.79% H; 11.10% N, 6.36% S. Found: 60.20% C; 4.75% H; 11.34% N, 6.69% S. Deviations most likely derive from residual solvent molecules.

3.4 Synthesis of α-methoxyethyl-ω-propargyloxypoly(ethylene glycol) (mPEG-prop)

Prior to synthesis, methoxypoly(ethylene glycol) (M_n= 350 g/mol) (6 mmol, 2.10 g) was dried at 50 °C in vacuum for 3 hours. Under nitrogen atmosphere, 10 mL dry dimethylformamide were added and the solution was cooled to 0 °C. Sodium hydride (720 mg of 60% dispersion in petroleum, 18 mmol) was added and the mixture stirred for 1.5 hours. Subsequently, propargyl bromide (18 mmol, 3.57 mL of a 80% solution in toluene) was slowly added and the resulting solution stirred for 48 hours at room temperature.
The reaction mixture was filtered before the solvent was removed in vacuum. The crude product was precipitated in petroleum ether, redispersed in tetrahydrofuran and then purified by filtration over silica gel (dichloromethane followed by methanol).

Yield: 690 mg

^1H NMR (DMSO-d_6, 300 MHz): δ [ppm]= 4.13 (d, ^4J= 2.4 Hz, 2H), 3.60-3.40 (m, polymer), 3.38 (t, ^4J= 2.4 Hz, 1H), 3.4 (s, 3H).
MALDI-TOF-MS (m/z): 357 $[M_n, n= 5 + Na]^+$, 401 $[M_n, n= 6 + Na]^+$, 417 $[M_n, n= 6 + K]^+$, 445 $[M_n, n= 7 + Na]^+$, 461 $[M_n, n= 7 + K]^+$, 489 $[M_n, n= 8 + Na]^+$, 505 $[M_n, n= 8 + K]^+$, 533 $[M_n, n= 9 + Na]^+$, 549$[M_n, n= 9 + K]^+$, 577 $[M_n, n = 10 + Na]^+$, 593 $[M_n, n= 10 + K]^+$, 621 $[M_n, n= 11 + Na]^+$, 665 $[M_n, n= 12 + Na]^+$.

3.5 Synthesis of PEG-PP

3-((2-Azidoethyl)thio)-*N*-(2-hydroxy-5-(1-(4-hydroxyphenyl)-3-oxo-1,3-dihydroisobenzofuran-1-yl)benzyl)propanamide (353 mg, 0.70mmol) and α- methoxyethyl-ω-propargyloxypoly(ethylene glycol) (116 mg, 0.3 mmol) were dissolved in 0.5 mL dimethylformamide. The solution was stirred at 110 °C for 60 hours. Afterwards, the solvent was removed in vacuum. For purification, column chromatography was performed. First, residual **PP-N3** was removed by use of acetone/dichloromethane (1:1), subsequent elution with methanol gave the pure product.

Yield: 317.90 mg

^{1}H-NMR (DMSO-d_6, 300 MHz): δ [ppm]= 9.88 (s, 1H), 9.66 (s, 1H), 8.32 (m, 1H), 8.10 (s, 1H), 7.89 (m, 1H), 7.84-7.76 (m, 1H), 7.73-7.59 (m, 2H), 7.19-6.90 (m, 4H), 6.85-6.67 (m, 3H), 4.67 (s, 2H), 4.61-4.43 (m, 3H), 4.15 (d, ^3J= 5.6 Hz, 2H), 3.60-3.38 (m, polymer), 3.23 (s, 3H), 2.96 (t, ^3J= 7.0 Hz 2H), 2.64 (t, ^3J= 7.2 Hz, 2H), 2.34 (t, ^3J= 7.2 Hz, 2H).
MALDI-TOF-MS (m/z): 729 [M_n, n= 2 + Na]$^+$, 773 [M_n, n= 3 + Na]$^+$, 795 [M_n, n= 4 + H]$^+$, 817 [M_n, n= 4 + Na]$^+$, 839 [M_n, n= 5 + H]$^+$, 861 [M_n, n= 5 + Na]$^+$, 883 [M_n, n= 6 + H]$^+$, 905 [M_n, n= 6 + Na]$^+$, 927 [M_n, n= 7 + H]$^+$, 949 [M_n, n= 7 + Na]$^+$, 971 [M_n, n= 8 + H]$^+$, 993 [M_n, n= 8 + Na]$^+$, 1015 [M_n, n= 9 + H]$^+$, 1037 [M_n, n= 9 + Na]$^+$, 1081 [M_n, n= 10 + Na]$^+$, 1125 [M_n, n= 11 + Na]$^+$.

3.6 Synthesis of 3-(3-(3-(prop-2-ynyloxy)-2,2-bis((prop-2-inyloxy)methyl)propoxy)- 2,2-bis((prop-2-ynyloxy)methyl)propoxy)prop-1-yne [2]

2.00 g (7.87 mmol) dipentaerythritol were baked out prior to synthesis. Under nitrogen atmosphere, 150 mL dry dimethylformamide were added and the resulting suspension was cooled to 0 °C. Then, sodium hydride (56.6 mmol, 2.27 g of a 60% suspension in petroleum) was added and the dispersion was stirred at 0 °C for an hour and then another two hours at room temperature. Afterwards, the mixture was cooled to 0 °C again and propargyl bromide (56.6 mmol, 6.10 g of a 80% dispersion in toluene) was slowly added. The reaction mixture was then stirred at 65 °C for 72 hours and the resulting solution was then poured on 100 mL ice and the aqueous phase was extracted with ethyl acetate. The combined organic layers were washed with saturated sodium hydrogen carbonate solution (2x) and brine (1x), dried with magnesium sulfate and subsequently; the solvent was removed in vacuum. The crude product was purified by flash chromatography (dichloromethane/hexanes 4:1).

Yield: 1.10 g (2.27 mmol, 58%)

^{1}H-NMR (500 MHz, $CDCl_3$): δ [ppm]= 4.09 (d, ^4J= 2.2 Hz, 12H), 3.49 (s, 12H), 3.35 (s, 4H), 2.39 (t, ^4J=2.3 Hz, 6H).
FT-IR [cm^{-1}]: 3292 (s), 2950, 2911, 2875 (m), 2118 (w), 1474,
1441 (w), 1090 (s).
ESI-MS (m/z(%))= 483.4 [M+H] (8) $^+$, 505.5 [M+Na]$^+$ (100), 527.4 [M+K]$^+$ (5).

3.7 Synthesis of mono-6^I-tosyl-6^I-desoxy-β-cyclodextrin [2]

β-CD (50.0 g, 44.0 mmol) was dissolved in 500 mL of a 0,4 M aqueous sodium hydroxide solution and cooled to 4 °C. While stirring vigorously, *p*-toluenesulfonyl chloride (35.0 g, 184 mmol) was added within 5 minutes. The resulting suspension was stirred for additional 30 minutes and was then filtered. The filtrate was neutralized by addition of hydrochloric acid and stirred for an hour. The precipitate that was formed thereby was isolated via filtration, washed with water (3x) and dried at 60 °C overnight.

Yield: 24.68 g (19.16 mmol, 43%)

^{1}H-NMR (500 MHz, DMSO-d_6): δ [ppm]= 7.77 (d, 2H), 7.45 (d, 2H), 5.74 (m, 14H), 4.79 (t, 7H), 4.50 (t, 7H), 3.18-3.78 (m, 42H), 2.45 (s, 3H).
FT-IR [cm^{-1}]: 3301 (b), 2924 (w), 1641 (w), 1554 (w), 1358 (w), 1021 (s), 836 (w).
MALDI-TOF-MS (m/z)= 1311.4 $[M+Na]^+$, 1327.5 $[M+K]^+$, 1465.5 [disubst. βCD + Na]$^+$.

3.8 Synthesis of mono-6^I-azido-6^I-desoxy-β-cyclodextrin [2]

Mono-6^I-tosyl-6^I-desoxy-β-cyclodextrin (10.0 g, 7.76 mmol) was dissolved in 100 mL water and heated to 80 °C. Subsequently, sodium azide (2.52 g, 38.8 mmol) was added and the mixture was stirred at 80 °C for 6 hours. Afterwards, the solution was filtered through a membrane filter (pore size 0.2 μm) and added to 600 mL acetone. The precipitate formed thereby was isolated and dried at 60 °C. Purification was performed via re-crystallization from water/acetone and subsequent drying at 60 °C.

Yield: 7.5 g (6,5 mmol, 85%)

^{1}H-NMR (500 MHz, DMSO-d_6): δ [ppm]= 5.74 (m, 14H), 4.92 (d, 1H), 4.85 (d, 6H), 4.49 (m, 6H), 3.65 (m, 28H), 3.32 (m, 14H).
FT-IR [cm^{-1}]= 3307 (b), 2922 (w), 2102 (s), 1644 (w), 1023 (s).
MALDI-TOF-MS (m/z): 1182.3 $[M+Na]^+$.

3.9 Synthesis of per-βCD-dipentaerythritol (DPE-CD) [2]

3-(3-(3-(Prop-2-inyloxy)-2,2-bis((prop-2-inyloxy)methyl)propoxy)-2,2-bis((prop-2-ynyloxy)methyl)propoxy)prop-1-yne was modified with mono-6^I-azido-6^I-desoxy-β-cyclodextrin according to a similar method described in literature [3].
3-(3-(3-(Prop-2-inyloxy)-2,2-bis((prop-2-inyloxy)methyl)propoxy)-2,2-bis((prop-2-ynyloxy)methyl)propoxy)prop-1-yne (13.9 mg, 29.0 μmol), mono-6^I-azido-6^I-desoxy-β-cyclodextrin (302.0 mg, 260 μmol), sodium-L(+)-ascorbate (4.5 mg, 23 μmol) and copper(II)sulfate pentahydrate (2.7 mg, 11 μmol) were dissolved in 8 mL dimethylformamide and placed in a pressure resistant test tube. The microwave-assisted reaction was performed under the following conditions: max. power: 100 W, max. temperature: 110 °C, max. pressure: 15 bar, reaction time: 50 minutes. The average conditions were: av. power: 68 W, av. temperature: 110 °C, av. pressure: 1.7 bar.
The reaction mixture was then added to 200 mL acetone, and the precipitate formed thereby was isolated, washed with acetone and dialyzed (mw cut off: 3.5 kD) against water. The product was obtained by freeze- drying.

Yield: 120 mg (16 μmol, 55%)

^{1}H-NMR (500 MHz, DMSO-d^6): δ [ppm]= 7.98-7.90 (m, 6H), 5.90-5.60 (m, 105H), 5.07 (b, s, 6H), 4.95-4.72 (m, 46H), 4.68-4.25 (m, 57H), 4.01-2.91 (m).
FT-IR [cm^{-1}]: 3305 (m), 2925 (w), 1414 (m), 1332 (m), 1153 (m), 1079 (s), 1025 (s).
MALDI-TOF-MS (m/z): 6312 (61) [M(diazide)+H]$^+$, 7448 (100) $[M+H]^+$.

4 ROESY spectrum of the PEG-PP–DPE-CD complex

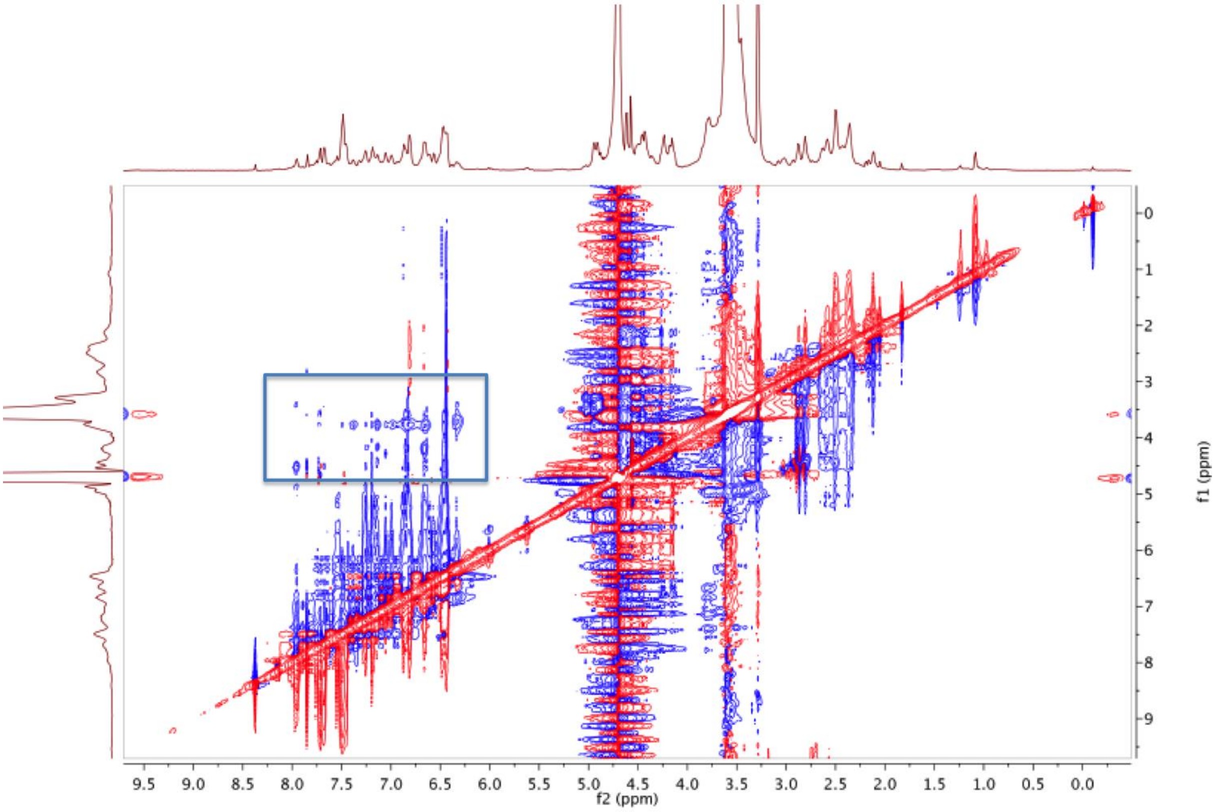

Figure S1. ROESY spectrum of **PEG-PP** (20 mg) and **DPE-CD** (18 mg) in 0.1 M NaOD solution (0.7 mL).

5 References

1. Fleischmann, C.; Ritter, H. *Macromol. Rapid. Commun.* **2013**, *34*, 1085-1089.
2. Müller, B.-K., Suprastrukturen modifizierter Polysaccharide durch hydrophobe Wechselwirkungen in Wasser. Ph.D. Thesis, Heinrich-Heine-University Düsseldorf, Germany, 2010.
3. Munteanu, M.; Choi, S.; Ritter, H. *Macromolecules* **2008**, *41*, 9619-9623.
